# Work Difficulties in People with Multiple Sclerosis

**DOI:** 10.1007/s10926-023-10149-9

**Published:** 2023-11-03

**Authors:** Michela Ponzio, Jessica Podda, Elena Pignattelli, Anna Verri, Benedetta Persechino, Bruno Kusznir Vitturi, Paolo Bandiera, Tommaso Manacorda, Matilde Inglese, Paolo Durando, Mario Alberto Battaglia

**Affiliations:** 1 Scientific Research Area, Italian Multiple Sclerosis Foundation (FISM), Genoa, Italy; 2 Italian Workers’ Compensation Authority (INAIL), Rome, Italy; 3 Department of Health Sciences, University of Genoa, Genoa, Italy; 4 Italian Multiple Sclerosis Association (AISM), Genoa, Italy; 5 Department of Neurosciences, Rehabilitation, Ophthalmology, Genetics, Maternal and Child Health (DiNOGMI), Center of Excellence for Biomedical Research (CEBR), University of Genoa, Genoa, Italy; 6 Occupational Medicine Unit, IRCCS Ospedale Policlinico San Martino, Genoa, Italy; 7 Department of Life Science, University of Siena, Siena, Italy; 8 Department of Research, Italian Multiple Sclerosis Foundation, via Operai 40, 16149 Genoa, Italy

**Keywords:** Multiple sclerosis, Work difficulties, Employment, Disability

## Abstract

**Purpose:**

This study identifies potential predictors of unemployment and describes specific work difficulties and their determinants in a subgroup of employed people with multiple sclerosis (PwMS). The specific work difficulties were evaluated using a validated tool that measures the impact of respondents’ symptoms and of workplace features.

**Methods:**

A cross-sectional study was carried out in Italy during 2021–2022. The subjects included were adults (18–65 years) with a diagnosis of MS, currently employed or unemployed. Logistic regression models were used to determine the association between each potential determinant and employment status, while linear regression models were used to determine the association between determinants and specific work difficulties.

**Results:**

The main risk factors associated with a higher risk of being unemployed were being older, living in the South of Italy/islands, and having a higher disability level, while protective factors against unemployment were having a high level of education and ‘stable’ employment (an open-ended contract). Fatigue was found to be associated with all work difficulties analyzed; mood disorders emerged as the main predictors of mental health-related work difficulties; level of disability and comorbidity significantly impacted physical health-related ones, and a good quality of life was found to improve both workplace-related and mental health-related difficulties at work.

**Conclusion:**

Identifying the most significant difficulties is a crucial step in the development of vocational rehabilitation interventions tailored to maximize the ability of PwMS to handle their job-related duties and demands.

## Introduction

Multiple sclerosis (MS) is a chronic inflammatory and degenerative disease of the central nervous system, affecting around 2.8 million people worldwide including one million in Europe and 127,000 in Italy [1]. MS is mainly diagnosed in young adults aged between 20 and 40 years, and it has an unpredictable but chronic and progressive course; it is the primary cause of non-traumatic disability in young adults in the Western world [2].

MS has a profound impact on the working lives of those affected because its early onset coincides with a period of life in which they may be struggling to enter the working world, or striving to develop professional skills and careers, and having to engage in complex relationships with coworkers [3].

The impact of MS on employment status has been evaluated in several studies carried out in different parts of the world. Most (90%) individuals diagnosed with the disease have worked prior to the diagnosis and approximately 60% are working at the time they are diagnosed; however, only a relatively small percentage (20–40%) continue to work after the diagnosis [4]. A European study showed that between 31 and 65% of people with MS (PwMS) are in employment [5], this rate depending on many factors, such as their degree of disability, disease duration, level of education, and type of work [6, 7]. As reported by Vitturi and colleagues in a recent review, unemployment and early retirement due to MS remain highly prevalent, despite a slight decline in the last decade, and their prevalence varies globally [8].

It is well known that MS, due to the age at onset and the fact that it reduces patients’ ability to enter or remain in the workforce, is associated with a heavy economic burden. Sobocki and colleagues, evaluating the total cost of MS in Europe based on data from nine countries, estimated that over 20% of it was attributable to production loss due to early retirement [9]. Similarly, in Italy, production loss emerged as the main contributor to the economic burden of MS, accounting, on average, for close to 30% of the total cost. The disease forced 1 in 3 employed PwMS to reduce their working hours and induced about 27% to change their type of work; over 60% of all these patients consequently saw their income reduced by over 30% [10].

Innovative treatments introduced over the past 20 years (specific drugs capable of preventing a significant percentage of relapses and delaying progression) have made it possible to better control the disease and achieve lasting reductions in physical disability [11]. This, in turn, has allowed a greater percentage of PwMS to continue working efficiently.

Several clinical, therapeutic and occupational factors have been identified as predictors of job loss or retention in PwMS. Factors associated with job loss or reduced capacity for work include the presence of a progressive form of MS, a long disease duration, problems of fatigue, movement-related symptoms, difficulty using the arms and hands, cognitive deficits, mood disorders, and type of immunomodulatory therapy [12–16]. Factors associated with job retention, on the other hand, include lower severity of symptoms and adequate management of drug therapies [17], but also work-related factors, such as level of education, having flexible working hours, having insurance coverage, being able to get to and from work and to access the workplace itself, and type of work [12, 15, 17]. A recent review reported that characteristics of the job, features of the work environment, social relationships at work, “negative work events”, and lack of information can all constitute barriers to work for PwMS [18]. Psychological and emotional factors also have been described as determinants of job retention in multiple sclerosis. Ford and colleagues reported self-efficacy was shown to be a strong mediator for work instability and is highly linked with self-management skills. It can contribute to a person’s confidence to raise problems with their managers and to successfully negotiate required workplace adjustments [19]. As things stand, the working status of PwMS has improved thanks to the clinical improvements obtained with disease-modifying drug (DMD) treatments, better symptom management, and more widespread application of support regulations such as, in Italy, the laws on the recognition of disability (Italian laws 118/71 and 104/92) and on targeted employment (Italian law 68/99). Nevertheless, critical issues remain.

As mentioned, MS is a disease that directly influences the professional lives of those affected, leaving them vulnerable to a spectrum of negative consequences, ranging from reduction of working hours to unemployment [20]. It has also been suggested that work demands in the context of worsening and unpredictable disease symptoms can be detrimental to mental health [21]. A proper understanding of difficulties at work as a function of both MS symptoms and features of the working environment could help to guide vocational rehabilitation (VR) interventions, VR being “a process whereby those disadvantaged by illness or disability can be enabled to access, maintain or return to employment or other useful occupation” [22].

There is evidence that VR, by tailoring job demands to an individual’s capacities, can improve work and health outcomes for people with long-term neurological conditions, like MS [23]. Chiu et al. (2013) found that the rate of MS participants successfully employed after receiving VR services was 48% [24]. While job retention and employment participation are clearly complex issues in MS, they offer scope for potential interventions and developments. Activities, such as risk analysis and management, aimed at identifying and addressing predictors of early exit from work, are fundamental in the context of prevention strategies aimed at improving the workplace inclusion of PwMS, and ensuring respect for their fundamental rights, including the right to accessibility, health and safety in the workplace. All this suggests that PwMS need holistic support in understanding and managing their condition, identifying workplace accommodations, and managing employers’ expectations through education.

## Aim

The aim of this study was to identify potential predictors of unemployment and to describe specific work difficulties and their determinants in a subgroup of employed PwMS. Specific work difficulties have been identified using a validated tool that measures the impact of respondents’ symptoms and of workplace features. Identifying the most significant difficulties is a crucial step both in the development of VR interventions designed to maximize the ability of PwMS to handle their job-related duties and demands, and also in verifying the results of interventions.

## Materials and Methods

A cross-sectional study was carried out in Italy during 2021–2022. The subjects included were adults (> 18 years) with a diagnosis of MS, currently employed or unemployed (aged ≤ 65 years and were thus of working age in Italy).

Participants were recruited among PwMS in contact with the Italian Multiple Sclerosis Association (AISM) through its social media channels. After clicking on a link, they were first asked to give their informed consent to their anonymous participation in the study.

Having consented, subjects completed an anonymous self-assessment online questionnaire collecting socio-demographic, clinical and work-related information with the help of various validated scales as described below.

Among clinical variables investigated the presence of the main comorbid conditions in MS, [25] namely, hypertension, diabetes, hyperlipidemia, chronic lung disease, autoimmune diseases, and cancer. A subsample of currently employed subjects also filled in a questionnaire about work difficulties (Multiple Sclerosis Questionnaire for Job Difficulties). The online questionnaire was delivered using the Survey Monkey® platform. The time required to complete the questionnaire was on average 15–20 minutes.

### Scales

The Multiple Sclerosis Questionnaire for Job Difficulties (MSQ-Job) evaluates work difficulties [26]. It is composed of 42 items forming six subscales and an overall scale, and it gives a total score ranging from 0 to 100. Higher scores indicate more severe problems. The questionnaire is intended to capture difficulties with work-related tasks as perceived by the person with MS, and it considers both MS-related (physical and mental) problems and workplace-related ones: in detail, two subscales (“*tactile perception and fine movement*” and “*movement and fatigue-related body functions*”) address physical health-related difficulties that affect work-related activities; another two (“*fatigue-related mental functions and symptoms*” and “*psychological and relational aspects*”) address mental health-related aspects, and the last two (“*time and organization flexibility in the workplace*” and “*company’s attitudes and policies*”) look at difficulties due to features of the workplace as perceived by the person with MS.

The Self-Expanded Disability Status Scale (self-EDSS) measures subjects’ level of disability. It is a descriptive scale derived from the original EDSS [27] and the patient-assessed Patient Determined Disease Steps (PDDS) scale [28]. As reported in the literature the EDSS compiled by patients was shown to be a very reliable self-reported measure [29].

A visual analog scale (VAS) ranging from 0 (no fatigue) to 10 (severe fatigue) was used to evaluate the impact of fatigue on work capacity as assessed by the participants.

The Hospital Anxiety and Depression Scale (HADS) is a well-established instrument for identifying anxiety and depressive symptoms [30]. It has 7 questions for anxiety and 7 for depression. For each question, subjects choose one out of four possible answers with scores ranging from 0 to 3. Total subscale scores thus range from 0 to 21, both for anxiety and for depression. Based on norms, a score of 0–7 is normal, 8–10 borderline abnormal, and 11–21 abnormal. Anxiety and depression were deemed clinically significant in the presence of subscale scores ≥ 8 [31]. HADS has been validated in Italian and for use in MS [31, 32].

The Multidimensional Scale of Perceived Social Support (MSPSS) measures individuals’ perceptions of support received from three sources: family, friends, and a significant other [33]. It has a total of 12 items, with 4 items assessing each source. Subjects indicate their level of agreement/disagreement, from 1 = very strongly disagree to 7 = very strongly agree. The total score is calculated by adding together the scores of the single items and dividing by the items included in the scale. A higher score represents higher perceived support. The MSPSS has been validated in Italian and found to show good internal consistency and it has already been used in MS [34, 35]. EuroQoL 5-Dimension 3-Level (EQ-5D-3 L) measures quality of life [36] and consists of two sections. In the first, the descriptive system includes five single-item dimensions of health-related quality of life (mobility, self-care, usual activities, pain/discomfort, and anxiety/depression). Each dimension is measured on a 3-point scale, and a single weighted score—the utility index—is obtained from the five questions. This score lies on a scale in which full health has a value of 1 and death has a value of 0, although negative values are allowed. In the present analysis, the EQ-5D-3 L results were translated into utility index values of between 0 (death) and 1 (full health), with negative values counting as 0. Utilities were estimated using the tariff developed by Scalone based on individuals from the Italian general population [37].

### Statistical Analysis

Socio-demographic, clinical, work-related variables and information collected with the help of validated scales (MSQ-Job, HADS, MSPSS and EQ-5D-3 L) are reported using descriptive statistics: frequencies and percentages for categorical variables; mean and standard deviation (SD) and median and interquartile range (IQR) for continuous variables.

Logistic regression models were used to determine the association between each potential determinant and employment status, while linear regression models were used to determine the association between determinants and specific work difficulties.

In the logistic regression models, the dependent variable (y) was categorized as 0/1 (currently employed/unemployed). In the linear regression models, work difficulties (y) were analyzed in three main domains: physical health-related, mental health-related, and workplace-related difficulties. The physical health-related score was calculated considering the items of the MSQ-Job related to “*tactile perception and fine movement*” and “*movement and fatigue-related body functions*”; the mental health-related score was calculated considering those related to “*fatigue-related mental functions and symptoms”* and “*psychological and relational aspects*”; while the workplace-related score included the items related to “*time and organization flexibility in the workplace*” and “*company’s attitudes and policies*”.

In all models, the potential determinants analyzed were: socio-demographic variables (age, sex, level of education and civil status), clinical variables [i.e., duration of illness, disease course, disability level (EDSS score), presence of comorbidity, anxiety and depression (HADS subscales) fatigue and treatment with DMD], social support (MSPSS), quality of life (EQ-5D-3 L utility score), and work-related variables [years worked, type of employment contract (fixed term or open-ended), type of employment (full-time or part-time), and type of work (categorized as light or heavy on the basis of the degree of flexibility, more vs. less respectively, that the worker likely enjoys)].

Independent variables found to be significant (p < 0.20) in the univariate analyses were included in a multivariate model using a stepwise backward model selection process with a pre-specified significance threshold of p < 0.05. The multivariate regression model was checked for multi-collinearity. A variable was eliminated if the variance inflation factor was 10 or over, or if the tolerance limit was less than 0.1. Analyses were performed using Stata Version 17 (StataCorp, College Station, TX).

## Results

### Sample Characteristics

A total of 422 PwMS consented to participate in this study. Of these, 75 (17.8%) were excluded for failure to meet the inclusion criteria or because they returned incomplete questionnaires, and 7 (2%) because they had never worked. The analysis was thus restricted to 340 PwMS: 244 employed and 96 unemployed. The characteristics of the sample are reported in Table 1.

**Table 1:** Socio-demographic, clinical and work-related variables in the study sample and univariate analysis of determinants of unemployment

Variables		Total sample N = 340	Employed N = 244	Unemployed N = 96	Single models OR (95% IC), p value
* Socio-demographic variables *					
Age in years, mean (SD) Range (min-max)		47.3 (10.3) (21–67)	45.7 (9.1) (21–67)	51.0 (12.1) (24–65)	1.05 (1.03–1.08), p < 0.001
Gender, n (%)	Female	234 (68.8%)	171 (70.1%)	63 (65.6%)	1
	Male	106 (31.2%)	73 (29.9%)	33 (34.4%)	1.23 (0.74–2.03), p = 0.425
Level of education, n (%)	Primary school	43 (12.7%)	23 (9.4%)	20 (20.8%)	1
	High school	165 (48.5%)	111 (45.5%)	54 (56.3%)	0.56 (0.28–1.11), p = 0.095
	University	132 (38.8%)	110 (45.1%)	22 (22.9%)	0.23 (0.11–0.49), p < 0.001
Civil status, n (%)	Living alone	132 (38.8%)	91 (37.3%)	41 (42.7%)	1
	Married/cohabitant	208 (61.2%)	153 (62.7%)	55 (57.3%)	0.80 (0.49–1.29), p = 0.357
Area of residence, n (%)	North	217 (63.8%)	163 (66.8%)	54 (56.3%)	1
	Center	61 (17.9%)	46 (18.9%)	15 (15.6%)	0.98 (0.51–1.90), p = 0.962
	South/Islands	62 (18.2%)	35 (14.3%)	27 (28.1%)	2.33 (1.29–4.20), p = 0.005
*Clinical variables*					
Duration of illness in years, mean (SD)		14.6 (9.9)	13.9 (9.4)	16.4 (10.9)	1.02 (1.00-1.05), p = 0.042
Disease course, n (%)	RR	248 (72.9%)	189 (77.5%)	59 (61.5%)	1
	SP/PP	92 (27.1%)	55 (22.5%)	37 (38.5%)	2.16 (1.30–3.58), p = 0.003
EDSS score, mean (SD)		3.4 (2.0)	3.1 (1.8)	4.3 (2.0)	1.39 (1.23–1.58), p < 0.001
Presence at least one comorbidity, n (%)		138 (40.6%)	96 (39.3%)	42 (43.8%)	1.20 (0.74–1.93), p = 0.457
Fatigue (VAS 0–10), mean (SD)		5.8 (2.5)	5.5 (2.5)	6.5 (2.3)	1.19 (1.07–1.32), p = 0.001
Currently treated with DMD, n (%)		285 (83.8%)	217 (88.9%)	68 (70.8%)	0.30 (0.17–0.55), p < 0.001
Presence of clinically significant anxiety, n (%)		177 (56.2%)	122 (54.2%)	55 (61.1%)	1.33 (0.81–2.18), p = 0.266
Presence of clinically significant depression, n (%)		116 (36.8%)	74 (32.9%)	42 (46.7%)	1.79 (1.08–2.94), p = 0.023
*Support from family, friends, and a significant *					
MSPSS score, mean (SD)		4.3 (1.2)	4.4 (1.1)	4.1 (1.2)	0.80 (0.65–0.98), p = 0.034
*Quality of life *					
*EQ-5D-3 L utility score, mean (SD)		7.7 (1.8)	8.0 (1.5)	7.0 (2.3)	0.74 (0.64–0.85), p < 0.001
* Work-related variables *					
Years worked, mean (SD)		21.3 (11.4)	21.0 (10.3)	21.9 (13.8)	1.00 (0.99–1.03), p = 0.513
Employment contract, n (%)	Fixed-term	88 (27.2%)	47 (20.5%)	41 (43.6%)	1
	Open-ended	235 (71.8%)	182 (79.5%)	53 (56.4%)	0.33 (0.20–0.56), p < 0.001
Type of employment, n (%)	Full-time	206 (63.8%)	151 (65.9%)	55 (58.5%)	1
	Part-time	117 (36.2%)	78 (34.1%)	39 (41.5%)	1.37 (0.84–2.25), p = 0.208
Type of work, n (%)	Heavy	101 (31.3%)	63 (27.5%)	38 (40.4%)	1
	Light	222 (68.7%)	166 (72.5%)	56 (59.6%)	0.56 (0.34–0.93), p = 0.024

*SD* standard deviation; *OR* odds ratio; *IC* interval confidence; *DMD* disease-modifying drug; *RR* relapsing-remitting, *SP/PP* secondary progressive/primary progressive; *VAS* visual analog scale; *MSPSS* Multidimensional Scale of Perceived Social Support; *EQ-5D-3 L* EuroQoL 5-Dimension 3-Level; *EQ-5D utility score transformed to allow changes to be measured in units of 1 as opposed to 0.1, for greater sensitivity; heavy work = manual worker, self-employed, director, official, nurse or health professional, cashier and shop assistant; light work = freelance, teacher, clerical worker

### Determinants of Unemployment

The univariate analysis identified older age, living in the South of Italy/islands, a longer disease duration, a progressive disease phenotype, a high EDSS score, and clinically significant depression as factors associated with a higher risk of being unemployed, while a high level of education, receiving DMD treatment, better social support (high MSPSS score), a good quality of life (high EQ-5D-3 L score), an open-ended employment contract, and light work were associated with a lower risk of being unemployed (Table 1).

Multivariate analysis confirmed some main variables, in particular, older age (OR = 1.06, p = 0.001), living in the South of Italy/islands as opposed to the North of Italy (OR = 3.24, p = 0.001), and a higher level of neurological disability (OR = 1.27, p = 0.003), as independently associated with a higher risk of unemployment, while having a higher level of education (OR = 0.46, p = 0.015) and an open-ended employment contract (OR = 0.25, p < 0.001) decreased this risk.

### Work Difficulties

Work difficulties were analyzed, using a specific questionnaire, in a subsample of participants, namely those currently in work. Of these working participants, 10 were excluded for not completing the MSQ-Job, leaving 234 for inclusion in this in-depth analysis.

Table 2, in addition to the scores for the three main domains, gives those for each of the six subscales. Our sample recorded higher scores for physical health-related work difficulties (median score 24.0, IQR: 11.5–40.4) particularly on the *movement and fatigue-related body functions* subscale (Table 2).

**Table 2:** Work difficulties measured using the MSQ-Job

Work difficulties	Median (IQR) score
*Physical health-related*	24.0 (11.5–40.4)
Tactile perception and fine movement	16.7 (8.3–33.3)
Movement and fatigue-related body functions	28.6 (14.3–50.0)
*Mental health-related*	20.8 (10.0–36.7)
Fatigue-related mental functions and symptoms	22.2 (11.1–36.1)
Psychological and relational aspects	16.7 (4.17–37.5)
*Workplace-related*	19.6 (8.9–37.6)
Time and organization flexibility in the workplace	14.3 (0.0–32.1)
Company’s attitudes and policies	25.0 (10.7–46.4)

### Determinants of Work Difficulties

#### Physical Health-Related Work Difficulties

Among the socio-demographic variables evaluated in the univariate analyses, only age showed a significant association (*β* = 0.60, p < 0.001) with physical health-related work difficulties; conversely, nearly all the clinical variables were associated with these difficulties, as were mood disorders (anxiety and depression). Higher social support (MSPSS score) and quality of life were associated with lower physical health-related difficulty scores (*β* = − 2.90, *p* = 0.014 and *β* = − 8.43, p < 0.001, respectively), while years worked (*β* = 0.46, p < 0.001) and part-time work (*β* = 7.52, *p* = 0.007) increased physical health-related difficulties at work.

The multivariate analysis confirmed that clinical variables, such as high disability (β = 3.72, p < 0.001) and level of fatigue (*β* = 1.99, p < 0.001), were associated with greater physical health-related difficulties, while good quality of life reduced these difficulties (*β* = − 4.01, p < 0.001) (Table 3).

**Table 3:** Univariate and multivariate analysis by three main domains of work difficulties

		Physical health-related		Mental health-related		Workplace-related	
		Single models	Overall model	Single models	Overall model	Single models	Overall model
* Socio-demographic variables*		β (SE), p value					
Age in years		0.60 (0.13), < 0.001		– 0.01 (0.13), 0.993		0.04 (0.17), 0.813	
Gender (Female)		– 2.30 (2.78), 0.408		– 0.81 (2.51), 0.746		0.79 (3.26), 0.809	
Level of education (Primary school)	High school	0.85 (4.56), 0.852		1.28 (4.13), 0.757		3.85 (5.36), 0.473	
	University	– 3.11 (4.55), 0.494		0.12 (4.12), 0.978		2.07 (5.35), 0.698	
Civil status (Living alone)	Married/cohabitant	4.81 (2.61), 0.067		0.73 (2.37), 0.758		5.59 (3.06), 0.069	
Residence area (North)	Center	4.20 (3.31), 0.205		0.32 (3.00), 0.915		– 1.31 (3.89), 0.737	
	South/Islands	6.49 (3.62), 0.074		4.16 (3.28), 0.206		5.91 (4.26), 0.166	
*Clinical variables *							
Disease duration in years		0.55 (0.13), < 0.001		0.23 (0.12), 0.058		0.08 (0.16), 0.611	
Disease course (RR)	SP/PP	12.36 (2.95), < 0.001		1.17 (2.76), 0.673		2.27 (3.59), 0.528	
EDSS score		7.30 (0.51), < 0.001	3.72 (0.59), < 0.001	2.13 (0.61), 0.001		1.69 (0.81), 0.038	
Presence at least one comorbidity		10.79 (2.51), < 0.001	5.13 (1.73), 0.003	3.10 (2.34), 0.187		2.81 (3.05), 0.358	
Fatigue		4.77 (0.39), < 0.001	1.99 (0.42), < 0.001	3.06 (0.41), < 0.001	1.23 (0.42), 0.004	2.87 (0.56), 0.001	2.43 (0.61), < 0.001
Treated with at least one DMD		– 4.72 (4.11), 0.252		0.96 (3.72), 0.797		– 2.02 (4.83), 0.676	
*Mood disorders*							
Presence of clinically significant anxiety		11.83 (2.49), < 0.001		20.57 (1.99), < 0.001	10.48 (2.12), < 0.001	9.76 (3.08), 0.002	
Presence of clinically significant depression		12.72 (2.65), < 0.001		21.88 (2.11), < 0.001	11.78 (2.26), < 0,001	18.94 (3.09), < 0.001	14.69 (3.20), < 0.001
*Support from family, friends, and a significant other *							
MSPSS score		– 2.90 (1.17), 0.014		– 5.49 (1.04), < 0.001		– 4.15 (1.40), 0.003	
*Quality of life *							
*EQ-5D-3 L utility score		– 8.43 (0.66), < 0.001	– 4.01 (0.72), < 0.001	– 5.63 (0.70), < 0.001	– 1.82 (0.76), 0.018	– 4.63 (0.99), < 0.001	
*Work-related variables *							
Years worked		0.46 (0.13), < 0.001		0.03 (0.12), 0.820		0.17 (0.15), 0.272	
Employment contract (Fixed-term)	Open-ended	– 1.00 (3.41), 0.769		– 4.08 (3.08), 0.187		– 3.18 (4.00), 0.427	
Type of employment (Full-time)	Part-time	7.52 (2.76), 0.007		4.12 (2.53), 0.105		2.55 (3.29), 0.439	
Type of work (Heavy)	Light	– 0.14 (3.01), 0.963		– 1.56 (2.72), < 0.001		– 5.75 (3.51), 0.103	

*SE* standard error; *DMD* disease-modifying drug; *RR* relapsing-remitting, *SP/PP* secondary progressive/primary progressive; *the EQ-5D utility score was transformed to allow changes to be measured in units of 1 as opposed to 0.1, for greater sensitivity; heavy job = manual worker, self-employed, director, official, nurses and health professional, cashier and shop assistant; light job = freelance, teacher, clerical worker. Text in parentheses refers to the reference category

#### Mental Health-Related Work Difficulties

None of the socio-demographic variables evaluated in the univariate analyses showed a significant association with mental health-related problems, while among the clinical variables, only disability and fatigue level were associated (*β* = 2.13, *p* = 0.001 and *β* = 3.06, p < 0.001, respectively) with mental health-related difficulties. Mood disorders (anxiety and depression) were strongly associated with mental health-related difficulties, while higher social support (MSPSS score) and quality of life were associated with lower scores on both the mental health-related difficulties subscales. Among the work-related variables, light work was associated with better mental health (*β* = − 1.56, p < 0.001).

The multivariate regression model confirmed a strong impact of mood disorders (anxiety, *β* = 11.29, p < 0.001 and depression, *β* = 12.21, p < 0.001, respectively) on mental-health related work difficulties, and a less strong effect of higher fatigue (*β* = 1.23, *p* = 0.004); these difficulties were less marked in the presence of good quality of life (*β* = − 1.82, p = 0.018).

#### Work Place-Related Work Difficulties

For workplace-related work difficulties, the univariate analysis highlighted the same potential predictors observed for mental health-related difficulties: disability (*β* = 1.69, *p* = 0.038) and fatigue (*β* = 2.87, *p* = 0.001), mood disorders (anxiety, *β* = 7.76, *p* = 0.002 and depression, *β* = 18.94, p < 0.001, respectively), social support (*β* = – 4.15, *p* = 0.003), and quality of life (*β* = – 4.63, p < 0.001). The multivariate model confirmed high fatigue level (*β* = 2.43, p < 0.001) and presence of clinically significant depression (*β* = 14.69 p < 0.001) as potential determinants of work difficulties.

## Discussion

This study set out to evaluate employment status in a sample of PwMS and continued with an in-depth analysis of employed MS subjects, investigating potential predictors of specific work difficulties. This last point was the novelty of the study.

### Determinants of Unemployment

In agreement with the literature, the main risk factors associated with a worse occupational outcome were an older age, living in the South of Italy/islands, and a higher disability level [20], while protective factors against unemployment were a high level of education level and ‘stable’ employment (an open-ended contract). Overall, the main determinants (age, educational level, residential area and open-ended contract) of unemployment were common in the Italian general population [38].

A higher level of education is associated with better job opportunities and a greater likelihood of being employed at all, and with better employment benefits and higher wages [39]. Accordingly, we found that more highly educated patients were more likely to be employed than patients with less education. In addition to economic benefits, higher education offers social advantages including a sense of control, social standing, and a greater influence over one’s own working conditions [40].

Our finding that area of residence was a risk factor for unemployment confirms that Italy’s economy is geographically divided, more so than any that of other country in Europe [41]. Evident regional differences in the country can be traced back to the historical divide between the South and the North, and are probably to be attributed to the uneven spread of industry, which is concentrated in the North.

Having an open-ended contract was the second protective factor against unemployment identified in our study, while having a fixed-term contract, associated with possible difficulties finding a new job when it ends, was found to increase the likelihood of unemployment.

We, like others [7], found no gender difference in unemployment, which suggests that women’s roles in the workforce may have changed over time, and reduced their relative vulnerability to unemployment. Others observed that male gender remains a risk factor for unemployment in MS [42].

### Determinants of Work Difficulties

In the second step of the study, we analyzed specific work difficulties in the subsample of currently employed PwMS. In this subsample, physical health-related difficulties were the ones found to impact the most on work activities. They included problems related to ‘*tactile perception and fine movement*’ (i.e., difficulties using a computer, difficulties with fine motor skills, physical impairments affecting the hands/arms, sensitivity to warmth/cold, physical impairments affecting the legs/feet) and to ‘*movement and fatigue-related body functions*’ (i.e., difficulties related to: movement, coordination or balance impairment, dizziness, problems with prolonged standing, getting easily tired, bowel problems, and fatigue). These aspects, as reported in literature, are known to be barriers to employment, and mobility disability in general is one of the most widespread and impactful consequences of MS [19].

Our analysis seeking to identify the potential determinants of specific work difficulties showed the physical health-related ones to be affected by clinical aspects (such as comorbidity and high levels of disability and fatigue) related to progressive worsening of neurological function, symptoms and motor deficits, which, in turn, can affect occupational performance [43]. Ambulation is one of the main aspects of disability measured by the EDSS, and a previous study found that physical problems with the legs or feet, and with the arms or hands, were among the main reasons for difficulties in the workplace [42]. As for the presence of comorbidities, it seems logical to assume that additional medical conditions exacerbate the global burden of illness and further impair the individual’s capacity to work. Patients with comorbidities have also been shown to have an increased risk of early retirement [44]. The high level of fatigue associated with physical health-related difficulties is a reflection of problems due to ‘*movement and fatigue-related body functions*’. Similarly, in the literature, fatigue measures were correlated with capacity to work and work limitations, and with the choice to work less work, switch to a different type of work, or leave work [45]. Moreover, we confirmed the relationship between professional life and quality of life observed in other studies, in particular worse health and physical function factors (i.e., worse overall health, less mobility) and a lower QoL [46].

With regard to mental health-related work difficulties, our study showed them to be clearly related to the presence of clinically significant anxiety and depression, confirming in general that depression is associated with reduced work participation (e.g., a longer time to return to work and a greater risk of unemployment), and to common mental disorders, including both depression and anxiety, leading to problems in work performance [47]. With regard to possible interrelations between depression and employment, a dynamic relationship can be hypothesized, with depression negatively impacting work performance, and difficulties at work or job loss being risk factors for depressive symptoms [48]. Similarly, in other studies anxiety negatively affected cognitive processing performance [49, 50], and it is possible to conclude that stronger levels of anxiety reinforce cognitive difficulties at work. As with depression, and given the common co-occurrence of depression and anxiety in people with MS, it seems reasonable to hypothesize a reciprocal influence [51], where difficulty fulfilling job requirements induces even more stress and anxiety, further interfering with cognitive performance.

These findings highlight the prominence of mental health-related difficulties in the workplace. In line with previous studies [52], our results show that depression and anxiety symptoms are important contributors to occupational difficulties. As seen with physical health-related difficulties, fatigue was found to be a relevant factor contributing to mental health-related ones, too. These included, as expected, problems strictly related to ‘*fatigue-related mental functions*’ (difficulties in understanding, with memory, in learning new tasks, in pronouncing specific words, visual disturbances, feeling sad, depressed, anxious or overly worried about not being productive or efficient, and difficulties with sleeping). The fatigue construct has been divided into motor, psychosocial, and cognitive or mental dimensions [53]. Cognitive fatigue significantly impairs daily life and is just as debilitating as motor fatigue to PwMS. In line with others [54], we found fatigue to be correlated with cognitive difficulties in PwMS, with effects on work performance.

Another clear relationship emerging from our study is that between mental health-related work difficulties and poorer quality of life, and it highlights a probable influence of quality of life on occupational performance and vice versa. Our results support previous research [43] on quality-of-life perception in which the associated physical and mental factors were the very ones that most significantly influence the occupational performance of PwMS.

Finally, the workplace-related difficulties analyzed in our study included problems related *to time and organization flexibility in the workplace* (lack of flexibility in working hours, having to work shifts or frequently work overtime, and difficulty securing a part-time role, taking breaks during working hours, getting paid leave, modifying tasks, role or working hours, or obtaining the possibility to work at home/remotely) and others related to the *company’s attitudes and policies* (lack of knowledge and understanding of disability and workplace rules, of the workplace rights of workers with disabilities, of national disability laws, and of the disease and its symptoms in the workplace, difficulty obtaining psychological support, poor relations with the employer, lack of understanding and appreciation, lack of career growth and uncertain or inadequate financial perspectives and/or social security coverage). The presence of clinically significant depression was found to be the main determinant of these difficulties. We hypothesized that situations that did not allow subjects to perform their roles adequately or that perhaps created impediments that made their MS more difficult to manage (no time/organizational flexibility or negative attitudes and policies on the part of the company) were a source of stress and may cause high levels of depression in PwMS. Efforts to increase employers’ awareness of the need for workplace adjustments to accommodate individuals’ needs and allow them to manage their symptoms effectively are needed, as are specific government-funded initiatives to encourage appropriate adjustments. Similar findings were reached in a previous study where depression was found to have a marked impact on psychological and relational aspects of work [54]. As mentioned, a dynamic relationship between depression and workplace-related variables can be hypothesized, with depression negatively impacting these variables, and difficulties in the workplace potentially causing depressive symptoms. The fatigue factor was also associated with these difficulties. Fatigue may indeed influence a number of very diverse parameters, not only physical but also emotional, as noted in previous work suggesting that fatigue has a significant impact on health perception and social functioning [55]. To overcome some barriers to work, approaches such as job crafting (i.e., fewer tasks or changing the way a task is conducted) and job carving (i.e., customizing a role by removing or swapping duties from and between roles) are recommended [56]. These approaches have been used to support people (such as the long-term unemployed) for whom getting back to work is a particular challenge, because they can increase the satisfaction of workers, making them feel valued, and improve productivity [23].

### Limitation

Given the retrospective and cross-sectional nature of the present study, it is difficult to accurately establish the temporal relationship between predictor variables and employment outcomes. Future longitudinal studies are needed to examine the suggested causal relations. Another limitation concerns the recruiting of subjects via the internet may be considered a problem although online questionnaires are the new frontier for data collection. Ascertainment is commonly assumed may be skewed when using web-based methods, as the technology may pose a barrier to the elderly, disadvantaged, technically inexperienced, or cognitively impaired [57]. For these reasons, the internet-using research participants may not be entirely representative of the MS population. Indeed, should not be underestimated the time required to complete the questionnaire (approximately 20 min) may hinder people who are actively working or who are both mentally and physically exhausted from participating. Furthermore, the enrollment source used, the social media channels of MS Society, reflects a greater distribution of its branches in the North of the country (due to historical and economic aspects) likely explaining the higher proportion of respondents in North Italy observed in the study. Some information was not collected in the study such as the type and start of treatment, which may be useful indeed patients who are early treated and with high-efficacy drugs are more advantageous in terms of disability and more likely to work efficiently. Finally, it should be remembered that the study assessed overall anxiety but not one due to fear for the future. Consistent evidence indicates that the unpredictability of the disease course (e.g., number of relapses over time) may increase mood disorders in MS patients [51, 58]. Since fear of relapse in the future could impact on MS patients about work, this aspect should be carefully investigated. Aspects of cognition like attention and concentration, information processing, and multitasking are all components critical in maintaining employment. Since cognitive problems negatively affect work, administering patient-reported instruments evaluating perceived cognitive deficits, like the Multiple Sclerosis Neuropsychological Screening Questionnaire (MSNQ) or the Perceived Deficits Questionnaire (PDQ), should be strongly recommended. Also, coping has previously been linked to employment status in PwMS. Avoidant coping styles, such as behavioral disengagement and denial, were associated with unemployment or a shorter time to unemployment. Additional research into coping and employment in MS is needed [21].

## Conclusion

In this study, we collected evidence using a tool, based on the biopsychosocial approach, that allows different elements (physical health, psychological/mental health and the work environment) to be taken into account when analyzing work difficulties among PwMS. MSQ-Job focuses on the specific domains in which workers with MS experience the most problems, and it considers both person-related and context-related issues. Once difficulties have been identified, tailored interventions aimed at maximizing the ability of PwMS to handle their work-related duties and demands can be planned.

Fatigue was found to be associated with all work difficulties; mood disorders emerged as the main predictors of mental health-related ones; some clinical aspects (level of disability, comorbidity) significantly impacted physical health-related difficulties, and a good quality of life was found to improve both workplace-related and mental health-related difficulties. Because it attacks the central nervous system, MS has a high probability of negatively affecting cognition, ambulation, coordination, and strength, and of causing fatigue, and any one of these impairments can negatively affect the performance of essential job functions, motivation to stay in work, and QoL. The vocational rehabilitation, through a multidisciplinary team composed of occupational therapists, physiotherapists, neuropsychologists, physicians, and nurses is able to manage the interaction between the impairments caused by MS, the physical environment, and the demands imposed by the work. A proper understanding of difficulties and their determinants at work could help to guide vocational rehabilitation interventions, in order to enable PwMS disadvantaged by illness or disability can be enabled to access, maintain or return to employment.

## Data Availability

The data that support the findings of this study are available on request from the corresponding author.
